# SUMO-Dependent Synergism Involving Heat Shock Transcription Factors with Functions Linked to Seed Longevity and Desiccation Tolerance

**DOI:** 10.3389/fpls.2017.00974

**Published:** 2017-06-13

**Authors:** Raúl Carranco, Pilar Prieto-Dapena, Concepción Almoguera, Juan Jordano

**Affiliations:** Departamento de Biotecnología Vegetal, Instituto de Recursos Naturales y Agrobiología de Sevilla, Consejo Superior de Investigaciones CientíficasSeville, Spain

**Keywords:** seed maturation, embryogenesis, longevity, desiccation tolerance, SUMO, HSFA9, HSFA4a

## Abstract

A transcriptional synergism between HaHSFA9 (A9) and HaHSFA4a (A4a) contributes to determining longevity and desiccation tolerance of sunflower (*Helianthus annuus*, L.) seeds. Potential lysine SUMOylation sites were identified in A9 and A4a and mutated to arginine. We show that A9 is SUMOylated *in planta* at K38. Although we did not directly detect SUMOylated A4a *in planta*, we provide indirect evidence from transient expression experiments indicating that A4a is SUMOylated at K172. Different combinations of wild type and SUMOylation site mutants of A9 and A4a were analyzed by transient expression in sunflower embryos and leaves. Although most of the precedents in literature link SUMOylation with repression, the A9 and A4a synergism was fully abolished when the mutant forms for both factors were combined. However, the combination of mutant forms of A9 and A4a did not affect the nuclear retention of A4a by A9; therefore, the analyzed mutations would affect the synergism after the mutual interaction and nuclear co-localization of A9 and A4a. Our results suggest a role for HSF SUMOylation during late, zygotic, embryogenesis. The SUMOylation of A9 (or A4a) would allow a crucial, synergic, transcriptional effect that occurs in maturing sunflower seeds.

## Introduction

The seed-specific heat-shock transcription factors (HSF) A9 and A4a (respectively, Almoguera et al., 2002; Tejedor-Cano et al., 2014) function in enhancing longevity and desiccation tolerance of seeds (Prieto-Dapena et al., 2006, 2008; Personat et al., 2014). Among the specific effects of A9 and A4a, this pair of HSF synergically activates transcription from small *Heat Shock Protein* (*sHSP*) gene promoters (Tejedor-Cano et al., 2014). A9 and A4a directly interact with each other through their oligomerization domains, an interaction that facilitates the nuclear retention of A4a by A9, which is required for the synergism (Tejedor-Cano et al., 2014). Transcriptional activation by A9 and A4a is repressed by the Aux/IAA protein HaIAA27 (Carranco et al., 2010; Tejedor-Cano et al., 2014). Stabilized forms of HaIAA27 (Carranco et al., 2010) and a dominant-negative form of A9 that incorporated the SRDX trans-repression motif (Tejedor-Cano et al., 2010)have been used to corroborate by loss-of-function the involvement of A9 in seed-longevity. This work indirectly indicated the contribution of additional -class A- HSFs, among them A4a as confirmed by subsequent work (Tejedor-Cano et al., 2010; Personat et al., 2014; Tejedor-Cano et al., 2014).

Post-translational modification has been found important in modulating transcription factor function, which in turn has profound effects on gene expression and many developmental programs in animals and plants. Lysine modifications that include acetylation, ubiquitination, methylation and, SUMOylation -in particular- have been found to be very relevant (Freiman and Tjian, 2003; Verger et al., 2003; Hill, 2015). SUMOylation, for example, modulates the activity of transcription factors involved in abiotic stress responses in plants (Lois et al., 2003; Miura et al., 2007, 2009; Cohen-Peer et al., 2010; reviewed by Castro et al., 2012). SUMOylation was also found to be essential for zygotic embryogenesis in seeds (Saracco et al., 2007). SUMOylation involves the covalent (and reversible) attachment of small ubiquitin-like modifier (SUMO) proteins to lysine. In the model plant Arabidopsis four different SUMO are expressed (SUMO1-3, and SUMO5). Mutational analysis in Arabidopsis has revealed that at least SUMO1/2 and SUMO3/5 do not have overlapping functions (Saracco et al., 2007; reviewed by Lois, 2011). All plants have at least one gene that encodes one form of SUMO, and the forms similar to Arabidopsis SUMO1 and SUMO2 are considered to represent the ancestral SUMO protein that is characteristic of eukaryotes. SUMOylation involves the consecutive enzyme-catalyzed steps referred to as SUMO E1 activation, E2 conjugation, and E3 ligation. De-conjugation of SUMO is catalyzed by SUMO-specific proteases. The SUMOylation and de-SUMOylation enzymes that have been more extensively studied in animal systems are conserved in plants such as Arabidopsis, tomato and rice (Kurepa et al., 2003; Novatchkova et al., 2004, 2012; Lois, 2011). Analyses of protein SUMOylation in plants have been performed mainly in Arabidopsis. This has revealed the prevalence of transcription factors and other nuclear-localized regulator proteins among the targets of SUMO (Elrouby and Coupland, 2010; Miller et al., 2010; Lois, 2011; Elrouby et al., 2013; López-Torrejón et al., 2013; Park et al., 2013). However, in plants the known SUMO-modified proteins represent only a much smaller number of SUMO targets in comparison to mammals and yeast (reviewed by Flotho and Melchior, 2013). We note that only a fraction of the potential SUMO-modified proteins has been experimentally confirmed. Furthermore, the functional consequences for reported protein SUMOylation remains unknown in most cases for plant proteins (reviewed by Lois, 2011).

The finding of potential SUMOylation sites in both A9 and A4a, as well as precedents for the importance of SUMOylation of HSF in both animal (Goodson et al., 2001; Hong et al., 2001; Anckar et al., 2006; reviewed by Björk and Sistonen, 2010) and plant systems (Cohen-Peer et al., 2010) called our attention and induced us to performing the experiments reported here. We could directly confirm the SUMOylation of A9 and also provide indirect evidence for the SUMOylation of A4a. Furthermore, we demonstrate that the modification of either A9 or A4a (respectively, at lysine residues K38 and K172) is required for their synergic transcriptional activation. Our results connect SUMOylation with HSF function during late embryogenesis in plant seeds. Thus, SUMO-modified HSFs might be involved in enhancing functions as seed-longevity and desiccation tolerance.

## Materials and Methods

### Transient Expression Assays in Sunflower

Directed mutation of the putative SUMOylation sites of HaHSFA9 were made by megaprimer PCR-mutagenesis (see Chen and Przybyla, 1994; Carranco et al., 2010). In HaHSFA9m1, lysine 38 was mutated to arginine. Mutations were introduced by PCR of the plasmid pBI221-HaHSFA9 (Almoguera et al., 2002) with the mutagenic oligo 5′-GGTTCCTCTcTAATCTTCATCATC-3′ and 5′-ATGGCAGGAGTAGTAAAAGAGTTTG-3′. This PCR product was used as megaprimer for a second amplification of the same plasmid together with oligo 5′-TTGCACATTTCGACACTTCC-3′. This final PCR product, digested with *Sty*I and *Bgl*II, replaced the corresponding wild type fragment in pSK-HaHSFA9 (Almoguera et al., 2002) to obtain pSK-HaHSFA9m1. In HaHSFA9m2, lysine 206 was mutated to arginine. Mutations were introduced by PCR of the plasmid pBI221-HaHSFA9 with the mutagenic oligo 5′-AGAAAGAATCACACTTAgACAAGAGATC-3′ and 5′-TTGCACATTTCGACACTTCC-3′. This PCR product was used as megaprimer for a second amplification of the same plasmid together with oligo 5′-ATGGCAGGAGTAGTAAAAGAGTTTG-3′. This final PCR product, digested with *Sty*I and *Bgl*II, replaced the corresponding wild type fragment in pSK-HaHSFA9 (Almoguera et al., 2002) to obtain pSK-HaHSFA9m2. HaHSFA9m1 and HaHSFA9m2 were introduced into pBI221 vector for transient expression assays as described for pBI221-HaHSFA9. The double mutant HaHSFA9m3 has both lysines 38 and 206 mutated to arginine. To make pBI221-HaHSFA9m3, the *Eco*RI-*Eco*RI wild type fragment from pBI221-HaHSFA9m1 was replace for its mutant version obtained from pBI221-HaHSFA9m2.

Directed mutation of the putative SUMOylation site of HaHSFA4a was performed by Mutagenex Inc., starting from plasmid pBI221-HaHSFA4a (Tejedor-Cano et al., 2014). Mutant HaHSFA4am1 has lysine 172 (codon AAA) substituted by arginine (codon AgA) whereas in mutant HaHSFA4am2, glutamic 174 (codon GAA) was substituted by alanine (codon Gcg). Plasmids pBI221-HaHSFA4am1 and pBI221-HaHSFA4am2 were used in transient expression assays.

Transient expression assays in **Figure 4B** were performed in 21 dpa sunflower embryos, essentially as described (Díaz-Martín et al., 2005). The amounts of plasmid DNA (per DNA precipitate, used for five shots) were: 50 ng pBI221-A9, 15 ng pBI221-A9m1, 2.5 μg pBI221-A4a, 2.5 μg pBI221-A4am1, 2.5 μg pBI221-A4am2 (effectors), 5 μg of –1132(G4):LUC (Díaz-Martín et al., 2005) (reporter) and 1 μg of pBI221-Rluc. The total amount of plasmid DNA was adjusted (if necessary) with pBI221 to 8.5 μg.

For the assays in **Figures 3B**, **4C**, sunflower leaves were bombarded essentially as described (Tejedor-Cano et al., 2010). The amounts of plasmid DNA in **Figure 3B** (per DNA precipitate, used for five shots) were: 5 μg pBI221-A9, 5 μg pBI221-A9m3, 5 μg pBI221-HaIAA27 (Carranco et al., 2010) (effectors), 5 μg of -126(G1):LUC (Díaz-Martín et al., 2005) (reporter) and 1 μg of pBI221-Rluc. The total amount of plasmid DNA was adjusted (if necessary) with pBI221 to 16 μg. The amounts of plasmid DNA in **Figure 4C** were as in **Figure 4B** except for the amounts of effectors: 10 ng pBI221-A9, 2.5 ng pBI221-A9m1, 2.5 ng pBI221-A9m2, 2.5 ng pBI221-A9m3, 2.5 μg pBI221-A4a, and 2.5 μg pBI221-A4am1.

### *In Vitro* SUMOylation Assays

Plasmid pRSET A-A9ΔDBD was made by replacing the *Xho*I to *Bgl*II fragment of HaHSFA9 from plasmid pRSET A-HaHSFA9 (construct “I” in Díaz-Martín et al., 2005) by a *Bgl*II digested PCR fragment obtained by amplification of plasmid pGBT9-HaHSFA9ΔDBD (Carranco et al., 2010) with oligos 5′-GTTCATGGCAGGAGTAGTAAAAGAG-3′ and 5′-TTGCACATTTCGACACTTCC-3′. To make plasmid pRSETA-A4a, PCR amplification of plasmid pUC-HaHSFA4a (Tejedor-Cano et al., 2014) was performed with oligos 5′-GGTATATCTTGGTCAATGATGA-3′ and 5′-GTAAAACGACGGCCAGT-3′. The *Sac*I digested PCR product was introduced between the *Sma*I and *Sac*I sites of pBI221. The amplified A4a sequence, which does not include the 5′-UTR, was then released by restriction with *Bam*HI and *Kpn*I and inserted into *Bgl*II and *Kpn*I digested pRSET A vector (Invitrogen).

Proteins 6xHis:Xpress:A9ΔDBD and 6xHis:Xpress:A4a were expressed in *E. coli* from plasmids pRSET A-A9ΔDBD and pRSET A-A4a, respectively, and purified with resin IMAC Sepharose^TM^ 6 Flast Flow, GE Healthcare. *In vitro* SUMOylation assays were performed as described in García-Domínguez et al. (2008). Hundred nanogram of purified protein was used as the target. Reactions were started with 2 mM ATP and stopped with Laemmli buffer. Proteins were detected by Western blot with antibodies against 6xHis (GE Healthcare).

### *In planta* SUMOylation Assays

For *Nicotiana benthamiana* leaves infiltration assays, the HaHSFA9 and HaHSFA4a mutants were transferred from the pBI221 plasmids used in transient expression assays (described above) to the pUC SPYCE(M) vector (Waadt et al., 2008) as described for pUC SPYCE(M)-HaHSFA9 (Carranco et al., 2010) and then to the binary vector kanII-SPYCE(M) as described in Tejedor-Cano et al., 2014. Plasmids kanII-SPYCE(M)-HaHSFA9 (Tejedor-Cano et al., 2014), kanII-SPYCE(M)-HaHSFA9m1, kanII-SPYCE(M)-HaHSFA9m2, kanII-SPYCE(M)-HaHSFA9m3, kanII-SPYCE(M)-HaHSFA4a (Tejedor-Cano et al., 2014) and kanII-SPYCE(M)-HaHSFA4am1 express fusion proteins HaHSFA9:YFP^C^, HaHSFA9m1:YFP^C^, HaHSFA9m2:YFP^C^, HaHSFA9m3:YFP^C^, HaHSFA4a:YFP^C^, and HaHSFA4am1:YFP^C^, respectively.

Mutant HaHSFA4am1 was also fused to GFP and introduced in the binary vector pRCS2-nptII (Tzfira et al., 2005) as described for HaHSFA4a in Tejedor-Cano et al., 2014. Plasmids pRCS2-nptII-EGFP:HaHSFA4a, pRCS2-nptII-EGFP:HaHSFA4a (NESmut) (Tejedor-Cano et al., 2014) and pRCS2-nptII-EGFP:HaHSFA4am1 express fusion proteins GFP:HaHSFA4a, GFP:HaHSFA4amNES and GFP:HaHSFA4am1, respectively.

For AtSUMO1 expression, pUNI-AtSUMO1 (ABRC; Stock # U17495) was amplified by PCR with oligos 5′-GGAGATCTAGACATGTCTGCAAACCAGG-3′ and 5′-ATGAGAGCTCAGGCCGTAGCACCAC-3′ and cloned into the *Sac*I (blunted with T4 DNA polymerase) and *Xba*I (Klenow filled) sites of pBI221 vector. For AtSUMO3, pUNI-AtSUMO3 (ABRC, Stock # U83833) was amplified by PCR with oligos 5′-GGAGATCTAGGCATGTCTAACCCTCAAG-3′ and 5′-ATGAGAGCTCAAAGCCCATTATG-3′ and cloned into vector pBI221 as described for AtSUMO1. For the stabilized mutant AtSUMO1 Q90A the transgene was released from plasmid pSK-Tag3-SUMO1 Q90A (Budhiraja et al., 2009) by digestion with *Nde*I and *Xba*I, filled with Klenow and cloned into vector pBI221 as described for AtSUMO1. AtSCE1 was released from plasmid, pGST-AtSCE1 (García-Domínguez et al., 2008) by *Sma*I and *Nco*I digestion, filled with Klenow and cloned into the *Sac*I (blunted with T4 DNA polymerase) and *Sma*I sites of pBI221 vector. AtSUMO1, AtSUMO1 Q90A, AtSUMO3, and AtSCE1 were excised from plasmids pBI221-AtSUMO1, pBI221-AtSUMO1 Q90A, pBI221-AtSUMO3, and pBI221-AtSCE1, respectively, as *Hind*III-*Eco*RI fragments and introduced in the corresponding sites of the vector pBI121.

**FIGURE 1:**
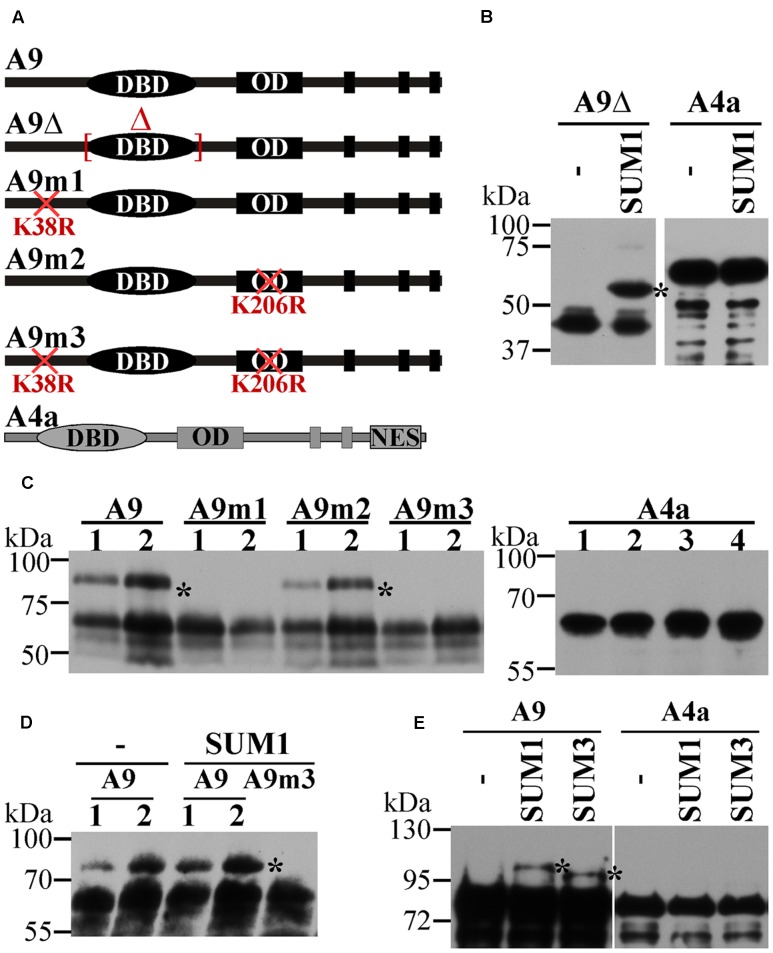
Direct evidence for the SUMOylation of A9. **(A)** Drawing of A9 and A4a forms used for the different SUMOylation assays. DNA binding domain (DBD), oligomerization domain (OD) and nuclear export signal (NES) are indicated. **(B)** *In vitro* assays using 6xHis:Xpress:A9Δ (A9Δ) or 6xHis:Xpress:A4a (A4a) fusion proteins with SUMO1 (SUM1) and without SUM1 added (–). **(C,D)** Assays performed in leaves of *N. benthamiana*. **(C)** Leaves were infiltrated with SUM1 and A9:YFP^C^ (A9), A9m1:YFP^C^ (A9m1), A9m2:YFP^C^ (A9m2), A9m3:YFP^C^ (A9m3) or A4a:YFP^C^ (A4a). **(D)** Leaves were infiltrated with A9:YFP^C^ (A9) or A9m3:YFP^C^ (A9m3) with (SUM1) or without (–) exogenous SUM1. Lane numbers indicate the different biological replicate results. **(E)** Assays in *E. coli* using Trx:6xHis:A9 (A9) or Trx:6xHis:A4a (A4a) fusion proteins with SUM1, SUMO3 (SUM3) or without SUMO proteins (–). Western blot detection are performed using anti-6xHis **(B,E)**, anti-HA **(C,D)** antibodies. Asterisks mark the SUMOylated form(s).

**FIGURE 2:**
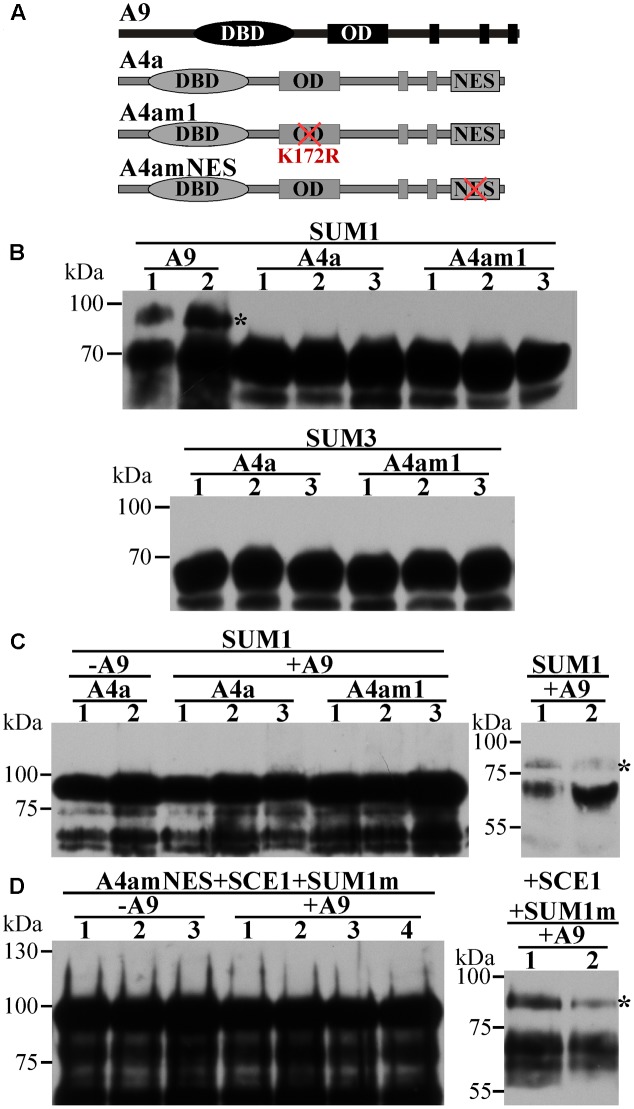
Further attempts to directly detect the SUMOylation of A4a in agroinfiltrated *N. benthamiana*. Western blot detection using the indicated fusion proteins and antibodies. **(A)** Schemes of the used WT and mutant A9 and A4a forms. Symbols are as in **Figure 1A**. **(B)** SUMOylation assays co-expressing SUMO1 (SUM1) or SUMO3 (SUM3) and the fusion proteins A9:YFP^C^ (A9), A4a:YFP^C^ (A4a) or A4am1:YFP^C^ (A4am1). A9 was assayed as positive SUMOylation control. **(C)** GFP:A4a (A4a) or GFP:A4am1 (A4am1) fusion proteins were not SUMOylated when coexpressed with SUM1 and A9 (+A9); coexpression without A9 (–A9), and detection of the SUMOylation of the co- expressed A9 (right panel) were used as negative and positive controls, respectively. **(D)**. The GFP:A4amNES (A4amNES) fusion protein was not SUMOylated when coexpressed with SCEI, and SUMO1Q90A (SUM1m). The same result was obtained in the absence (–A9) or presence (+A9) of coexpressed A9; however, the coexpressed A9 was SUMOylated (right panel). Anti-HA antibody was used to detect the fusion proteins in **B** and the right panels of **C** and **D**. Anti-GFP antibody was used to detect the fusion proteins in the left panels of **C** and **D**; for further details, see Section “Materials and Methods”. Rest of symbols as in **Figure 1**.

*Nicotiana benthamiana* leaves infiltration assays were described in Carranco et al. (2010). In **Figures 1C,D**, **2B–D** (right panels), Agrobacterium harboring plasmids expressing the fusion proteins HaHSFA9:YFP^C^, HaHSFA9m1:YFP^C^, HaHSFA9m2:YFP^C^, HaHSFA9m3:YFP^C^, HaHSFA4a:YFP^C^, or HaHSFA4am1:YFP^C^ were co-infiltrated with plasmid for expression of AtSUMO1, AtSUMO1 Q90A, AtSUMO3, or AtSCE1 as indicated in the corresponding figures. In **Figures 2C,D** (left panels), Agrobacterium harboring plasmids expressing the fusion proteins GFP:HaHSFA4a, GFP:HaHSFA4amNES, or GFP:HaHSFA4am1 were co-infiltrated with plasmid for expression of HaHSFA9:YFP^C^, AtSUMO1, AtSUMO1 Q90A, or AtSCE1 as indicated in the corresponding figures.

### SUMOylation Assays in *E. coli*

For HSFs expression in *E. coli*, HaHSFA9 was amplified from pBI221-A9 by PCR with oligos 5′-aaaaagcaggcttcATGGCAGGAGTAGTAAAAG-3′ and 5′-agaaagctgggTCAGCTTTTGGGATCAACTC-3′. HaHSFA4a was amplified from pBI221-A4a with oligos 5′-aaaaagcaggcttcATGATGAATGATGTTCATG-3′ and 5′-agaaagctgggTCACTTCTCTCTACTGAAG-3′. The resulting DNA fragments were cloned into pDONR201 and then transferred to pER32b-GW (that introduces N-terminal Trx and 6xHis tags), as described in Elrouby and Coupland (2010). Each resulting plasmid was transformed into *E. coli* BL21 (DE star) together with pCDF-SAE and pACYC-SCE-SUMO3 (Elrouby and Coupland, 2010) or pCDFDuet-AtSUMO1-AtSCE1 and pACYCDuet-AtSAE1-AtSAE2 (Okada et al., 2009). As negative control *E. coli* BL21 (DE star) was also transformed with similar plasmid combinations lacking the SUMO harboring plasmids. All the genes where induced over night with 0.5mM IPTGat 28°C.

### Western Blot Assays

Proteins from agroinfiltrated *N. benthamiana* leaves or *E. coli* cells were extracted with 2x Laemmli’s buffer. Total protein samples of *E. coli* (5 μg) or *N. benthamiana* leaves (40 μg) were run in SDS-PAGE: 8% acrylamide gels for GFP-fusion proteins and 10% for the rest. Anti-6xHis antibody (GE Healthcare) at 1/1000 dilution was used to detect 6xHis-tagged proteins. Anti-HA-Peroxidase antibody (Roche) at 1/1000 dilution was used to detect YFP^C^-fusion proteins (detection did not need a secondary antibody). Anti-GFP antibody (Clontech) at 1/2000 dilution was used to detect GFP-fusion proteins. Anti-mouse IgG-Peroxidase (Oncogene^TM^) at 1/5000 dilution was used as secondary antibody for Western blots with anti-6xHis. Anti-rabbit IgG-Peroxidase (GE Healthcare) at 1/50000 dilution was used as secondary antibody for Western blots with anti-GFP. The ECL Prime system (GE Healthcare) and X-ray films were used for detection of the recombinant proteins.

### *In planta* Protein Localization Assays

*Nicotiana benthamiana* leaves were infiltrated with Agrobacterium harboring plasmids expressing the fusion proteins GFP:HaHSFA4am1 alone or together with HaHSFA9m3:YFP^C^ and analyzed with a confocal laser-scanning Olympus FV1000 microscope as described in Tejedor-Cano et al. (2014).

### Statistics

Detailed procedures for ANOVA analyses have been described previously (see Prieto-Dapena et al., 2006, and references therein).

## Results

### SUMOylation of A9 and A4a: Direct Detection of SUMOylated A9

Using the SUMOplot^1^ and SUMOsp 2.0 (Ren et al., 2009) programs, we identified two putative SUMOylation sites in A9 (K38, K206), while a single site (K172) was present in A4a (**Table 1A**). Site-directed mutagenesis of these sites (changing K to R) was used to analyze their potential SUMOylation. Diverse SUMOylation assays were accomplished using the WT HSFs and different mutant proteins (**Figure 1A**). For example, *in vitro* SUMOylation assays performed with the Arabidopsis SUMO enzymes E1, E2, and E3, plus SUMO1 and the recombinant A9Δ protein easily detected a band with retarded mobility consistent with the SUMOylation of A9. In contrast, parallel analyses using the WT A4a protein failed to detect SUMO1-modified forms of A4a (**Figure 1B**). To confirm *in vivo* the SUMOylation of A9, to identify the SUMOylated residue(s), and to further attempt detection of A4a SUMOylation, additional assays were made in *Nicotiana benthamiana* (**Figure 1C**). When plasmids encoding the A9 and Arabidopsis SUMO1 proteins were co-infiltrated in leaves, retarded mobility of the WT A9 protein was observed. A similar result was obtained after co-infiltration of SUMO1 with the A9m2 mutant form of A9. In contrast, SUMO-modified forms of A9 were not detected when the A9m1 (or A9m3) mutant proteins were similarly co-expressed. These results confirmed SUMOylation of the A9 protein, also suggesting that SUMOylation *in planta* occurs mainly at position K38, even without co-expressed SUMO1 (**Figure 1D**). The equivalent analyses using SUMO1 and the WT A4a protein did not reveal hints of SUMOylation (**Figure 1C**). We also unsuccessfully tried to detect A4a SUMOylation in *N. benthamiana* with different strategies that were designed to cope with several possible limiting steps either individually or combined (**Figure 2**). For example, we explored if SUMO3 is required instead of SUMO1, or if the SUMOylation of A4a needs A9 (**Figures 2B,C**, respectively). In addition, to examine the possibility that a very efficient SUMO de-conjugation is what prevents detection of modified A4a, we coexpressed A4a and a SUMO1 mutant form impaired in de-conjugation (SUMO1 Q90A, Budhiraja et al., 2009); again not achieving success (**Figure 2D**). We also used A4amNES a mutant form of A4a that is not exported from the nucleus (Tejedor-Cano et al., 2014). This form was tested with Arabidopsis E2 (SCE1) and SUMO1, SUMO3, or SUMO1 Q90A (**Figure 2D**, results for SUMO1 Q90A). We thus ruled out that nuclear localization of A4a is required for its SUMOylation. Additional SUMOylation assays were performed in *E. coli*. We used Arabidopsis SUMO1 or SUMO3, further attempting detection of the SUMOylation of A4a. Only A9 was SUMOylated in *E. coli*, and SUMOylation was observed using either the SUMO3 or SUMO1 form (**Figure 1E**). The results presented so far confirmed that A9 is SUMOylated *in planta* (at least at K38 and perhaps also at K206); while the SUMOylation of A4a (at K172), if real, it would be more elusive.

**Table 1:** SUMOylation prediction.

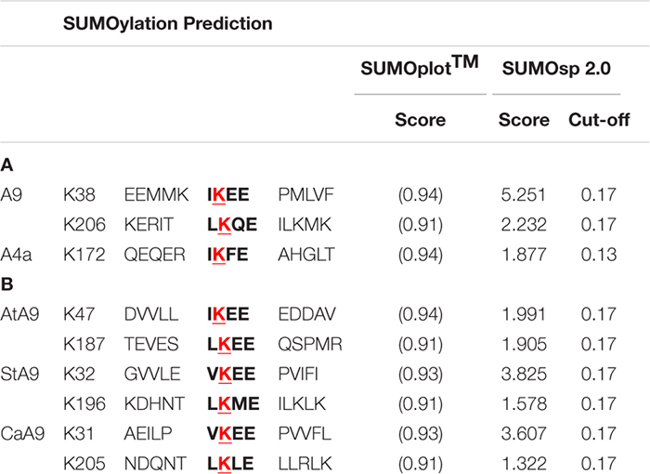

***(A)** SUMOplot^TM^ and SUMOsp 2.0 predictions for A9 and A4a. The predicted motifs showing higher probability for SUMOylation in each HSF protein are indicated in bold face and the predicted SUMOylated lysines underlined. **(B)** Examples of similar predictions for other, dicot plant, A9 HSFs: AtA9 (*Arabidopsis thaliana*, NP_200218); StA9 (*Solanum tuberosum*, XP_0006357708); and CaA9 (*Coffea arabica*, AFK24440). Compared to what predicted for A9 (in A), the putative SUMOylation sites are located at slightly different positions within the N-terminal extension of the HSFs (K31, K32, and K47), or in the oligomerization domain (K187, K196, and K205).*

### Functional Consequences of SUMOylation of A9 and A4a. Indirect Detection of SUMOylated A4a

**FIGURE 3:**
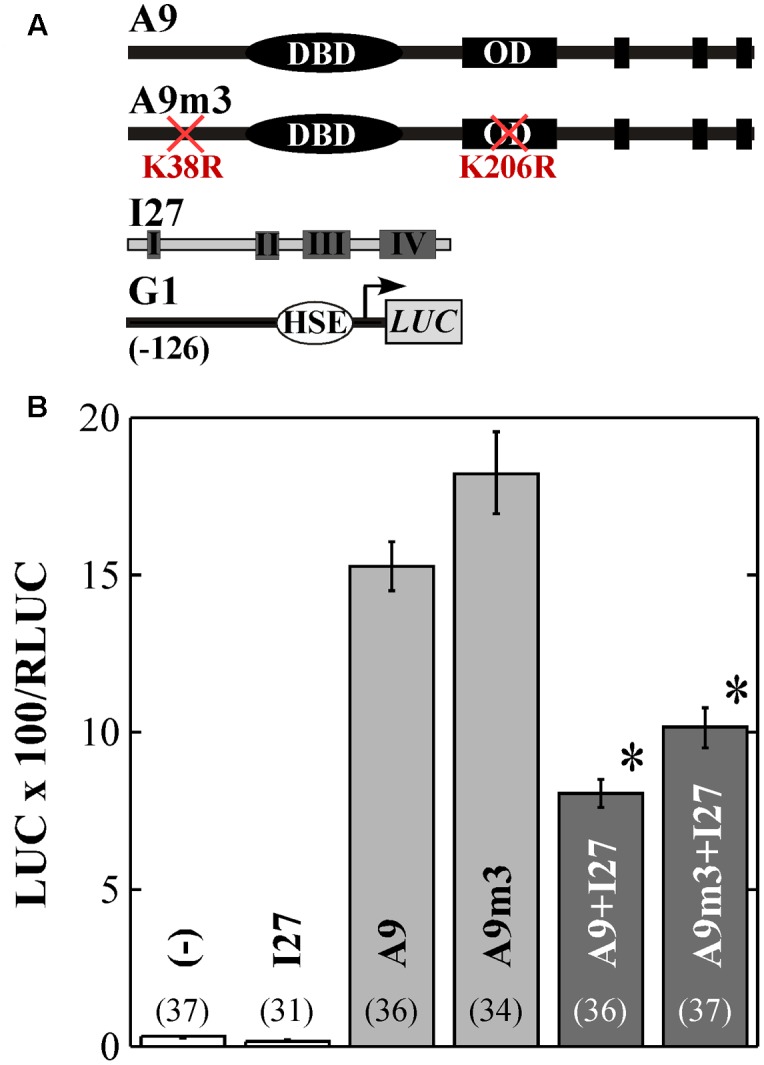
Mutation of K38 and K206 does not affect repression of A9 by HaIAA27 (I27). The *G1* reporter gene was used in transient expression assays performed using sunflower leaves bombarded with the indicated combinations of the effectors depicted in **(A)**. **(B)** Results obtained upon bombardment without (–) and with the indicated combinations of effectors. Numbers in brackets correspond to sample size. Bar shading and the asterisks indicate the similar repression by I27 of A9 and A9m3.

**FIGURE 4:**
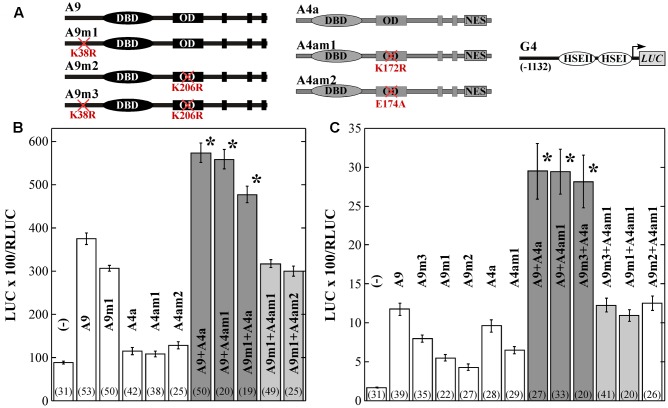
Requirement of K38 (in A9), or K172 (in A4a) for the synergism between A9 and A4a. Indirect evidence for the SUMOylation of A4a at K172. **(A)** Transient expression assays performed using the depicted *G4* reporter gene and effectors. **(B)** Results of the experiments performed with sunflower embryos. **(C)** Results of similar experiments performed in bombarded sunflower leaves are represented as in **(B)**; see also symbols for **Figure 3**. **(B,C)** dark bar shading and the asterisks indicate, significant, synergistic transcriptional effects and light bar shading indicates the loss of synergism.

To further investigate the occurrence and relevance of A9 and A4a SUMOylation, we performed transcriptional assays using the WT and mutant HSF forms (in separate or combined). Most precedents in literature link SUMOylation with the regulation of repression in both animal and plant systems (reviewed by Verger et al., 2003; Gill, 2005; García-Domínguez and Reyes, 2009). Thus, we started by using the A9 and A9m3 forms in transient repression assays by IAA27 (Carranco et al., 2010). We found that in bombarded sunflower leaves, the K38R and K206R substitutions in A9m3 moderately augmented the transcriptional activation of the *HaHsp17.6 G1* (G1, Carranco et al., 1999) promoter, about 1.2-fold compared to what observed with WT A9 (**Figure 3**). However, the A9 and A9m3 proteins accumulated to similar levels in infiltrated leaves of *N. benthamiana* (**Figure 1C**). The moderately enhanced transactivation capacity of A9m3 (**Figure 3B**), and that of other mutant forms of A9 (see **Figures 4B,C**), would fit precedent work performed with other mammalian and plant HSFs analyzed in separate (Anckar et al., 2006; Hietakangas et al., 2006; Tateishi et al., 2009; Björk and Sistonen, 2010; Cohen-Peer et al., 2010). We remark that the mutated lysines in A9m3 did not affect transcriptional repression by IAA27, which was observed to the same extent using either A9 or A9m3. (**Figure 3**; statistical analyses from these and other experiments reported here are included in the Supplementary Table S1). Thus, SUMOylation of A9 would not affect transcriptional repression of A9 by IAA27. IAA27 not only represses activation by A9, but also coactivation by A9 and A4a (Tejedor-Cano et al., 2014). Thus, we also performed additional transient experiments originally designed to explore the potential effects of SUMOylation on repression by IAA27 of the synergism between the two HSFs in bombarded sunflower embryos. These experiments where performed using the *Hahsp17.7 G4* promoter (G4, Almoguera et al., 1998). In these experiments (**Figure 4B**), the mutant A9m1 form activated transcription with higher efficiency than the WT form; this effect was compensated by adjusting the amounts of these HSFs (see Materials and Methods). A statistically significant synergistic effect was still observed when the mutant form of one HSF was combined with the WT form of the other HSF (**Figure 4B**). However, and surprisingly, the transcriptional synergism between A9 and A4a was fully abolished when the mutant forms of both HSF proteins were combined (**Figure 4B**). These results strongly suggest that SUMOylation of A9 at K38 (**Figure 1**) or modification of A4a at K172 is required for the synergism; because of this unexpected result it was not necessary further testing IAA27 in **Figure 4**. Similar effects of the mutant proteins on the synergism were observed by transient expression in bombarded sunflower leaves, where additional lysine substitutions (A9m2 and A9m3) in A9 were also analyzed. These experiments confirmed the major effect of SUMOylation at K38, also indicating a minor contribution of SUMOylation at K206 (**Figure 4C**). We did not directly detect SUMOylated A4a, but the similar lack of synergism with A9m3, obtained with the A4am1 (with a substitution of the lysine residue) and A4am2 mutants, provide a strong, although indirect, evidence for the SUMOylation of A4a at K172 (**Figure 4B**). The E174A substitution in A4am2 would impair interaction of A4a with the SUMO-E2-conjugating enzyme Ubc9 in the vicinity of K172 (see for example, Sampson et al., 2001). E174A is not expected to affect other modifications of K172 (as acetylation or ubiquitination). We therefore infer that the post-translational modification at K172 in A4a that is required for the transcriptional synergism is, most likely, also a SUMOylation.

### SUMOylation Does Not Affect Nuclear Retention of A4a

**FIGURE 5:**
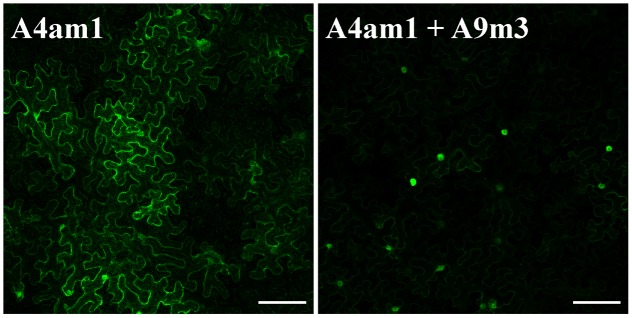
K38 and K206 (in A9) and K172 (in A4a) are not required for nuclear retention of A4a. Confocal images in *N. benthamiana* leaves. Left: A4am1 depicts a mainly cytosolic localization. Right: co-expression of A4am1 and A9m3 leads to nuclear localization of A4am1. Scale bars = 100 μm.

The transcriptional synergism involving A9 and A4a requires the mutual interaction of both HSF in the nuclei. In absence of A9, A4a is mostly localized in the cytosol; the interaction of A4a with A9 hinders a NES motif localized in A4a leading to nuclear retention of A4a (Tejedor-Cano et al., 2014). Therefore, we investigated if the lysine residues identified as SUMOylation sites in A9 and A4a are necessary for nuclear retention of A4a. The results in **Figure 5** show that the K172R mutant form of A4a showed a mostly cytosolic localization similar to what reported for WT A4a (Tejedor-Cano et al., 2014). Co-infiltration of the mutant forms of A4a and A9 (**Figure 5**) enhanced the nuclear localization of A4am1, again in a similar way as described for the co-expression of the two WT HSF (Tejedor-Cano et al., 2014). Therefore, major effects of the analyzed mutations on HSF hetero-oligomerization would be unlikely. We conclude that SUMOylation at the residues mutated in the two HSF proteins used in the experiments of **Figure 5** would not be required for their mutual interaction and for the subsequent nuclear retention of A4a.

## Discussion

The reported direct evidence for SUMOylation of A9 at K38 (**Figure 1**), and indirect evidence for SUMOylation of A4a at K172 (**Figure 4B**), adds these two HSFs to the -yet small- set of plant proteins that are known to be modified by SUMO (Budhiraja et al., 2009; Elrouby and Coupland, 2010; Miller et al., 2010, 2013; Lois, 2011; Castro et al., 2012; Elrouby et al., 2013; López-Torrejón et al., 2013; Park et al., 2011, 2013). HSFA9s from Arabidopsis and other dicot plants present potential SUMOylation sites at positions close to that of K38 and K206 in sunflower A9 (**Table 1B**). This does not occur for the sunflower A4a site and other A4a HSFs. Precedent studies in animal systems showed that, in general, SUMOylation enhances the function of a variety of repressor complexes (reviewed by Verger et al., 2003; Gill, 2005; García-Domínguez and Reyes, 2009; for example, see Kang et al., 2010). In contrast, the conjoint analysis of A9 and A4a uncovered a novel and unexpected positive effect for the SUMOylation of these two HSFs. Their synergic co-activation did not occur when the SUMOylated lysines were mutated in A9 and A4a (**Figure 4B**). This would fit less usual reports, where SUMOylation enhances transcription, also only in animal systems (Kotaja et al., 2002; Wang et al., 2004, 2007; Choi et al., 2011; Yang et al., 2012; Huber et al., 2013); even one of these reports showed that SUMOylation could enhance synergic interactions between transcription factors (Kotaja et al., 2002).

We explored simple mechanistic explanations for how the analyzed SUMOylations are required for the synergism. For example, the results in **Figure 5** showed that an effect of SUMOylation on the interaction between A4a and A9 is unlikely. SUMOylation might still enhance the interaction of A4a with A9, but this effect would be too-transient or subtle for it to be detected under the conditions in the experiments of **Figure 5**. The synergism would rather be affected by the analyzed mutations at a subsequent stage after mutual HSF interaction and nuclear co-localization (Tejedor-Cano et al., 2014). More complex, alternative explanations of two types would be compatible with the results reported here. SUMOylation has been shown to induce conformational changes in proteins as for example thymine DNA glycosylase (Baba et al., 2005; Steinacher and Schär, 2005). Protein–protein interactions, which in some instances enhance transcriptional activation, have been also shown to be induced by SUMOylation (Ishov et al., 1999; Kotaja et al., 2002; Wang et al., 2004; Choi et al., 2011). Thus, SUMOylation of A9 (or A4a) may facilitate a conformational change required for the synergism, and (or) interaction with a still non-identified coactivator. HSF coactivator proteins have been identified mostly in animals, as for example DAXX, ASC-2, and CHIP (Boellmann et al., 2004; Hong et al., 2004; Kim et al., 2005). In plants, HSF-coactivator studies include only work on HSFB1, which is involved in heat stress responses in tomato (Bharti et al., 2004). However, and as far as we know a connection between SUMOylation and HSF coactivation has not been explored besides the results reported here.

Our results are consistent with an activation model in which SUMOylated A9/A4a complexes bound to DNA sequentially recruit transcriptional coactivator(s) and (or) chromatin remodeling factor(s). We cannot exclude that A9/A4a SUMOylation may also induce conformational changes that, as shown for HSF1 and CHIP1 (Kim et al., 2005), could contribute to the proposed recruitment. We showed that HSF SUMOylation is required for a transcriptional synergism that is involved in the enhancement of two crucial functions in seeds (Tejedor-Cano et al., 2014, and references therein): longevity and desiccation tolerance, both acquired in maturing zygotic embryos (reviewed by Dekkers et al., 2015; Sano et al., 2016; Leprince et al., 2017). Precedent studies in plants have only indicated functional connections of SUMOylation with non-embryonic development, for example with the control of flowering (Xu and Yang, 2013; Elrouby et al., 2013; Elrouby, 2014). From the results reported here, we propose that seed HSF SUMOylation may also contribute to explaining the essential role of SUMO in seed development that was inferred from a previous study (Saracco et al., 2007). Our results reveal that, as in animals (see for example, Kang et al., 2010), SUMOylation is involved in the modulation of transcriptional activity in embryos.

## Author Contributions

RC, PP-D, and CA performed the experiments and analyzed the data; JJ designed the research and wrote the manuscript. All the authors agreed on the contents of the paper and declared no conflicting interest.

## Conflict of Interest Statement

The authors declare that the research was conducted in the absence of any commercial or financial relationships that could be construed as a potential conflict of interest.

## Footnotes

1 http://www.abgent.com/sumoplot
