# Supplementary material for: SUMO-Dependent Synergism Involving Heat Shock Transcription Factors with Functions Linked to Seed Longevity and Desiccation Tolerance
Source: Front Plant Sci. 2017 Jun 13;8:974. doi: 10.3389/fpls.2017.00974 (PMC5468958; doi:10.3389/fpls.2017.00974)
Supplement: Supplementary file 1 [file Table_1.PDF]

	Luc/Rluc±SE		
**TF combination**	**Co-bomb**	**Sum**	**ANOVA**
**A9 + A4a (Fig. 4B)**	573.48 ± 22.69	401.53 ± 13.41	**F=120.241, P=0.0001**
**A9 + A4am1 (Fig. 4B)**	558.49 ± 22.84	394.65 ± 13.41	**F=61.608, P=0.0001**
**A9m1 + A4a (Fig. 4B)**	477.26 ± 18.84	333.60 ± 7.17	**F=44.914, P=0.0001**
A9m1 + A4am1 (Fig. 4B)	316.78 ± 9.14	326.72 ± 7.17	F=0.387, P=0.5343
A9m1 + A4am2 (Fig. 4B)	299.46 ± 11.27	346.66 ± 7.17	F=5.868, P=0.0157
**A9 + A4a (Fig. 4C)**	29.48 ± 3.55	19.74 ± 0.82	**F=7.810, P=0.0054**
**A9 + A4am1 (Fig. 4C)**	29.41 ± 2.89	16.62 ± 0.79	**F=24.483, P=0.0001**
**A9m3 + A4a (Fig. 4C)**	28.15 ± 3.38	15.92 ± 0.82	**F=15.539, P=0.0001**
A9m3 + A4am1 (Fig. 4C)	12.27 ± 0.85	12.81 ± 0.47	F=1.995, P=0.1583
A9m1 + A4am1 (Fig. 4C)	10.93 ± 0.77	10.36 ± 0.44	F=0.063, P=0.8020
**A9m2 + A4am1 (Fig. 4C)**	12.50 ± 0.90	9.14 ± 0.44	**F=6.804, P=0.0093**

**I27 repression of A9: A9WT to A9m3 (Fig. 3B)**	F=0.376, P=0.5417

Table S1. Summary of statistical data for the different transcriptional effects observed by transient expression in sunflower. Top. Statistical analysis of the synergistic interactions observed by transient expression in sunflower. Data from the indicated TF combinations and Figures were analyzed following the logarithmic normalization and one-way ANOVA. We show the SE and mean reporter activity (Luc/Rluc) obtained after co-bombardment (Co-bomb) and from the sums of the activities separately induced by the corresponding TFs. Note that from each sum of two individual activity values, the basal level (activity without TF) was subtracted once. The statistic (F) and probability (P) values are shown for each comparison between the Co-bomb and Sum mean activities. Statistically significant differences (P< 0.01) are indicated with bold face. Bottom. ANOVA also confirmed that repression by I27 of A9 was not different from repression of A9m3.
